# Intermittent Fever, Part 2

**Published:** 1892

**Authors:** P.P. Wells


Merc-corr., Mur-ac., Natr-mur., Nit-ac., Nux-v., Op., Par., Petrol., Phos., Phos-ac., Puls., Ran-scel., Rhus, Sabad., Sarsap., Scill., Sep., Sil., Spig., Spong., Staph., Stann., Sulph., Thuja, Verat., Zinc.

*Before midnight.*—Amm., Arg., Aur., Calad., Carbo-an., China, Mur-ac., Phos., Puls., Rhod., Sabad., Sulph., Verat.

*About midnight.*—Ars., Caust., Hepar, Sil., Stram.

*After midnight.*—Amm-mur., Ars., Bor., Calad., Canth., Caust., Cham., Cocc., Dros., Ferr., Kali, Laur., Nitr., Nux-v., Petr., Phos., Ran-scel., Rhus, Scill., Sulph., Thuja.

*Daytime.*—Alum., Ant-crud., Ant-tart., Asar., Dros., Hell., Hepar, Kali, Natr., Rhod., Sabin., Sil., Spig., Sulph-ac., Verat., Viola-od.

Recurring at the same hour.—*See ante.*

*Evening, between four and eight o'clock.*—Bov., Graph., Hell., Hepar, Lyc., Magn-mur.

*Every fourteen days.*—Ars., Calc., Chin., Puls.

*Every year.*—Ars., Carbo-veg., Lach., Sulph.

Having the *time* of the first appearance of the first element of the paroxysm, we may proceed to consider that element, ascertain its character, and give it its true place in the group of phenomena, the simillimum of which we are about to seek, and find if we may. We may find this chill, heat, or sweat, or some other fact indicative of the disturbance of function or functions of forces and organs, which may prove controlling factors in the choice of this simillimum. This other fact may precede either of the usual elements of the paroxysm, and be followed by either after an interval of time of greater or less extent. After this precedent fact, if there be such, then note the order of appearance and succession of the ordinary elements, and also their symmetry of proportion of duration, or want of this, as well as the symmetry of intenseness of development of each, or the want of this; and in this last case, which element preponderates in violence. Note, also, any departure from the usual order of succession of the elements of the paroxysm, and also if either of these predominate over the others in violence, or if either be wanting altogether, or be only feebly present.

If there be symptoms preceding either of the three usual elements of the paroxysm these are to have the first consideration, and the remedies in the record of which these are found are to be noted, and that one which has them, with the greater number of the later symptoms, is to be selected. If the paroxysm be initiated by either of the three usual elements of the fever, then this initial element is to be the starting-point in our search for the specific. If this be, as is oftener than otherwise, the chill, then this is to be questioned as to its intensity and duration, its predominance as compared with the other elements of the paroxysm, its simple or complex character (*i. e.*, mixed with the heat or sweat). If complex, the character of this—*i. e.*, is the chill and heat, or sweat concomitant or alternating? Is the heat so mixed general or partial? Is it internal or external? Is it actual heat or only a sensation of heat? If partial, what are the parts so affected? If the combination be with the sweat, the inquiry is similar. Is the sweat general or partial? If partial, on what parts? Is the sweat hot or cold, profuse or but slight? Are the chill and the sweat concomitant, or alternating? If there be any peculiar phenomena developed with the sweat they are to be carefully noted. If the chill be mixed with both the other elements at the same time, then which of them is predominant, and if they are in alternation with each other, in what order of succession do they appear?

*Chill predominant.*—The remedies most strongly characterized by this fact are Acon., Agar., Alum., Amm-mur., Anac., Ant-crud., Ant-tart., Apis, Arn., Ars., Asaf., Asar., Aur., Bar., Bell., Bor., Bov., Brom., Bry., Calad., Calc., Camph., Cann., Canth., Caps., Carbo-an., Carbo-veg., Caust., Chel., Chin. Cic., Cina, Cocc., Colch., Coloc., Con., Creos., Croc., Cupr., Cycl., Dig., Dros., Dulc., Euphorb.

If after this general following of details for the gathering of the totality of the symptoms of a case of ague, and this being secured and written down, that it may be kept as a whole in our view, and after comparing this view with the general hints we have given for the just differentiation of drugs related to the group we have gathered, there be doubt as to the specific for the case, this must be resolved by reference to the repertory, contained in Bœnninghausen's peerless work on fevers already referred to.* Beginning with the first division, that of the circulation, let him look for the remedy which has affected the circulation of provers in the manner most similiar to its changed manifestations in the case to be prescribed for.† Treating the condition of the vessels, as to their fullness or anemic condition, in the general, or in individual parts of the organism; the pulse, as to its rapidity or slowness, fullness or emptiness, hardness or softness, and all the circumstances and conditions by which the pulse is affected in these or in any other ways; note sense of throbbing, or perceptible beating of arteries or veins, in general or in particular parts; note the action of the heart as to its violence or fullness, regularity or irregularity of its rhythm; the character of its beat—is it fluttering, intermittent, felt, audible, visible, trembling, etc., and also all circumstances and conditions by which these modifications of cardiac action are affected. Find the most similar facts in the repertory and note the drug which has produced them, and then pass to the second division.

*The chill.*‡—Is it general or partial? If partial, what are the parts affected? If it be of one side only, which side? Is the chill with shaking or shuddering, or with shivering or trembling? In what part did the chill first manifest itself, and in what direction did it progress from that point? By what circumstances or

* We had made considerable progress in translating the repertory as a constituent of this paper, before we saw a translation of the complete work by the very competent hand of Dr. Korndœrfer, of Philadelphia. We have no hesitation in saying this work should be in possession of every homœopathic physician, who does not read the original, if he is ambitious of the best possible success in treating agues homœopathically. To such a practitioner the work is indispensable, and above all price. On a first view of this masterpiece let no one be repelled or discouraged by the great wealth of material here before him, but let all who will bring to it honest industry and perseverance be assured that by a very brief use of it in searches for specifics, the gained familiarity will make it in their hands a power for good which will leave them in no need of resort to Quinine to cover their imbecility.

† For these symptoms, see *ante*, p. 12.

‡ For concomitant symptoms of chill, see *ante*, p. 16.

conditions is the chill produced or aggravated? What time in the twenty-four hours does it attack? Is it at the same hour it re-appears, or does it anticipate this hour, or is it at a later hour? Note all the concomitants of the chill, such as pains, the parts in which these appear, their character, what aggravates or relieves them—thirst, if present, how does drinking affect the chill? Is it before, during, or after the chill? Does it call for cold or warm drinks? Does the patient drink much or little at a time? Note gastric symptoms, as nausea, vomiting, eructations, etc. How is the chill affected by external warmth? Is it aggravated or relieved, or simply not relieved? Note the effect of the chill on the organs of sense, as of the eyes, as moving of objects before the eyes, flames, flimmerings, photophobia, mists, cloudy vision, obscured vision, lost vision, trembling before the eyes. In the ears—pains, pressure, heat, redness, sensitive to noise, ringing sound, rushing, deafness, etc. Note the face, its color; red, pale, one-sided redness, heat, coldness, sweat, convulsions, distortion, etc. Of the mouth—burning, dryness, offensive smell, salivation, coated tongue, etc. Appetite—aversion to food, disgust for food, hunger, desire for beer, etc. Taste in mouth—bitter, insipid, putrid, metallic, salt, acid, sweet, etc. Eructations—vomiting, bitter, bloody, of food, acid, slimy, black, watery. Nausea and waterbrash.

Pains in stomach, liver, spleen, kidneys, hypogastrium, distention and coldness in abdomen.

Diarrhœa—painful or painless, constipation, urgency to stool, and tenesmus.

Urine—urgency, fruitless urgency, frequent urination, painful, seldom, involuntary, retention.

Sneezing, coryza, fluent coryza, dry coryza.

Affections of respiration—suffocating attacks, deep inspiration. Breath hot, cold; slow respiration, loud rattling, quick, sighing, irregular.

Cough—with expectoration, or without. Hoarseness.

Pains in chest. Sensation of warmth, heart throbbing.

Pains in shoulder-blades, shootings in back and loins.

Pains in upper extremities, dead hands, swollen veins, blue hands, heat of hands, dead fingers, heat of fingers, blue nails.

Pains in lower limbs, hips, thighs, knees, legs, feet dead, feet swollen, heat of feet, cold feet.

Bodily exhaustion, swelling of veins, nervous excitability, limbs asleep, carphologia, insensibility of limbs, clonic cramps, tonic cramps, paralysis, muscular weakness, jerking of muscles, fainting, tearing pains in muscles, ditto in joints, ditto in hands, trembling and stretching of limbs, heaviness of limbs, shootings in the joints, stiffness of joints, bodily restlessness, as if bruised, trembling sensation, internal jerkings, contractions of limbs.

Blue color of skin, burning of skin, in ulcers, itchings, shootings, contraction of skin.

Yawning, sleepiness, sleeplessness, starting with fright in sleep, sliding down in bed in sleep, murmuring, snoring, talking, groaning in sleep.

The phenomena between the chill and the heat, if any, are to be carefully noted and studied. Then those of the heat, in like detail and carefulness as those of the chill, are to be noted, and especially its characteristics, circumstances, and concomitants. (See *ante*, concomitants of heat, pages 19–22.)

So also of the phenomena between the heat and the perspiration, if any, and the phenomena and concomitants of the sweating. (See *ante*, concomitants of sweating, pages 24–25.)

In prescribing for ague, the physician is more, if possible, than in treating any other disease, to keep in mind the *absolute* necessity of his gathering the "totality of the symptoms," and comparing the record of his drug with this, and so ascertaining its similarity before he accepts it as his remedy. If its record have not this similarity, it is no remedy for his case, though it may have cured thousands of other cases which have been called by the same name. Names will not, here or elsewhere, be accepted as in any degree a basis for specific prescribing. And herein, let it be remembered, is one of the fundamental differences between the antique school and ours in methods of dealing with the sick. The old-school prescriber is first concerned to find a *name* for his case, which he calls diagnosis, and then proceeds to give the drug which tradition or his own imagination has set up for its cure.

Not so the specific prescriber, and never so, when this, hitherto, opprobrium is before him to be cured.. Let him, if he be honest and honestly emulous of such success as is possible—*i. e.*, of the best, get into his mind the "totality" of the morbid phenomena of the case before him, and that no part of this "totality" escape him, let him familiarize himself with the idea of its possible extent, as shown in the schedule of symptoms and concomitants given in this paper. He may find of them many or few in the case before him, how many or few he can only know after he has gone over the entire ground of the four divisions of the phenomena of ague as here they are gathered from living examples of this many-sided plague. Let him have no shrinking away from the multitude of possibilities he may encounter, but seize whatever of these may be before him, make them all his own, and then proceed with patience and courage to search for the simillimum of these in the record of drugs before him, and be assured if he does this manfully and habitually in this and all sicknesses the habit will soon convert even the tyro into the master, and his life-work will become a work of joyful successes, and even the most extended group of symptoms will cease to be to him a tangled "wilderness," but rather under his hands will become an orderly range of facts, leading him by their light and their relations to the curative and the cure of this "plague of all diseases," just as in any other form of sickness. Let him not once think of the *name*, when the question is of the remedy, but only of this, it may be in the beginning a seemingly endless group of symptoms to him, but by practical familiarity with this multitude it will by and by cease to confuse, and become the guides to the prescriber God's law has ordained them to be. To cure ague by homœopathic means and methods is no more difficult than it is to cure other sicknesses by the same means and methods. The only difference is, there may be more facts requiring their simillimum in the curative, but this only gives to the master prescriber the greater certainty in his choice of his remedy, he having faithfully performed his duty in gathering and using his indispensable "totality." It should be always remembered that obtaining this is by far the most difficult part of a cure. In comparison with this, finding the remedy is often a trifle. But, because difficult, is it therefore to be neglected? If any one be tempted to pass over this first duty lightly, and to content himself with something less than this totality, it should be evidence to himself that he has mistaken his calling, as it will be to all capable of honest and intelligent homœopathic practice that he can never realize the success this promises to those who are more capable and faithful.

The object of this paper has not been to do the work of others who may be called to attempt the cure of this fever, but only to point to such of them as may be willing to give to the duty the time and labor obedience to our law demands, the way in which the work is to be done by themselves. There are, and have been, many ways of dealing with this problem of cure. For example, by use of a so-called universal specific, Cinchona, or some one of its elementary principles. It has been already seen that this proceeding can be no better than any other base empiricism—*i. e.*, giving medicines to sick men without reference to any fundamental principle of relationship of drugs and diseases, by which these are made curatives of the others, and that by reason of the great number and variety of the facts involved in the problem which go to make up the case to be prescribed for.

The absurdity of this resort will be still more apparent if we consider the only possible principle of relationship of curative between drugs and sicknesses, must be either that of similarity or of dissimilarity. It is apparent at the first glance that no one drug can, in its action, be similar to so many and so various facts, and to the infinite variety in the combination of these facts as they are met in our practical dealings with this fever. The difficulty is not lessened if we contend rather for the dissimilar principle, but is rather increased, not only for the reasons which apply to the similar principle, but because many of these facts have no dissimilar or opposite principle, or fact possible. For example, the headache and vomiting, and many others. This failure of possible applicability of this principle to the treatment of this fever demonstrates another fact—that it is not one of nature's laws of cure. Her laws are of universal applicability as to all their intended objectives. And so we are left to the one and only possible law of relationship, so far as our present knowledge enables us to see, the law of the similars. It has been our object to show how one is to proceed, under its guidance, to find the specific for each case for himself. So far is this specific from being found in any *one* drug or nostrum, it may be the next dozen cases one may treat may be found each to require a different remedy. Many have said and believed, apparently, that they could not cure ague by homœopathic means and methods. The difficulty with such, we believe, has been their attempt to cure *ague* rather than their *patient*. They found the defining symptoms of ague. This gave them their diagnosis, and on this they proceeded to attempt a cure. They acted on the facts required for their diagnosis, and left out of their consideration those, and perhaps *all* those required by therapeutics, and of course the result was *failure*. But this was not homœopathic practice, but only an attempt to practice Homœopathy on *allopathic* principles, and this nearly always is more or less of failure. The attempt is too much like an effort to mingle light and darkness. *Success* in the treatment of this fever can only be realized by the use of the simillimum to the "*totality*" of the symptoms, and this, when used, knows no *failure*.

The simillimum having been found, and taking it for granted that the prescriber is ambitious of the best possible results from its administration, the question will arise, how shall he use it that these may the more certainly be secured?

The question involves the potence, time of its administration, repetition, and form and quantity of its dose. As to potence, there has been more written and spoken on this than has been characterized by knowledge of the facts or principles involved in the subject. And too often it has happened that the excited feeling evinced has been in the exact inverse ratio of this knowledge. There is one rule, we believe, which may be accepted at the outset, and which, we think, dominates the selection of the potence. It is this: In direct ratio of the similarity of the recorded symptoms of the drug action, and those of the case to be cured, should the potence chosen be a *high* one—*i. e.*, the greater the similarity the higher should be the potence. It is but a general rule, and there may be circumstances in the vital condition of the patient which may at times render the rule practically not beneficial. For instance, if the susceptibility of the patient to drug action be *extreme*, as it sometimes is, medium potencies may be found to give better results than extremely high ones, even though the similarity be great. This may not be just as those might anticipate who regard potentization as a process of reduction, each higher possessing less power to affect the organism than those below it. This is not the fact, and the fact demonstrates the dynamic nature of that in the drug which cures. There need be no hesitation in selecting a higher number because of the presence of circumstances or influences which are supposed to endanger the operation of the dose so that its legitimate results may be lost in consequence of them. It is a mistake to regard these as more liable to disturbance from such causes than are lower numbers. Indeed, just the reverse of this is true, as has been observed in many instances by most competent men. The error which once regarded these high numbers as more readily affected by disturbing influences than are the lower, grew out of the thought that the higher were weaker than the lower as to power to resist adverse influences, and weaker because of assumed less of drug *matter* in them. The exceptions to the rule given for the selection of the potence are rare, though they are occasionally met. It may be remarked, by the way, that *violence* of symptoms are no reason for a departure from the general rule. Violence of diseased action is just as certainly met by high as by low numbers, and not unfrequently their use is attended by a much better success. Let the thought be dismissed entirely and forever that *strong* and *weak* are in any way expressive of the difference between *low* and *high* potencies.

Then, as to the *time* of giving the remedy? It should *not* be given just before the expected return of the paroxysm, for the reason that, being an agent which acts on the organism in a similar direction and manner as the acting morbid cause, it will be liable to intensify the violence of the paroxysm, and the more accurately it has been selected—*i. e.*, the more accurately the likeness has been recognized and appreciated—which constitutes the medicine a curative. It should not be given *during* the paroxysm, for the same reasons.*

As has been already suggested, there may arise, occasionally, in paroxysms, such violence of some of their elements as to render interference with this a duty, but the remedy for this violence is not often the simillimum for the case, so that the exception to the rule as to time of giving this is more seeming than real. The time for giving the simillimum is immediately *after* the close of the paroxysm.†

Then as to *repetition* of the dose. The practice has been different with practitioners of more or less authority. Some of these, who have been most skillful and successful, have given, immediately after the paroxysm, one dose, either dry or dissolved, and have left this to do its work, not permitting interference with this by repetitions or doses of other medicines. If the prescriber can always be *sure* of his selected remedy being an exact simillimum of his case, this would, no doubt, be the best method of procedure. But can he always be *sure?* Is it quite certain that an exact simillimum for *every* case, in the present state of our knowledge, is possible, even to the search of the most skillful? He will be either bold or reckless who affirms this. It is not difficult to conceive of the possibility of a case existing where the variety of the combinations of elements so numerous has presented us a case so made up as that

---

* Since this sentence was written we have read a paper on the homœopathic treatment of this fever which directs the selected remedy to be given *during* the paroxysm. We have no doubt this is bad advice, and, if acted upon, will often lead to intenser suffering of the patient, and not unfrequently to a defeat of the cure.

† If the interval of intermission between the paroxysms be very short, so that necessary time for the development of the action of the dose is not present, it may be necessary to anticipate the complete termination of the paroxysm, and give the dose before the sweating has entirely passed off.

its perfect likeness is not found in any one of our numerous proved medicines. Indeed, it is possible that the best the average practitioner may be able to do for his case may be to find a proximate likeness for it, by reason of want of knowledge, or of absence from our materia medica of any drug which has a *perfect* likeness to the case in its record. From this state of things has come the practice of giving the selected medicine immediately after the paroxysm, and repeating the dose every two, three, or four hours during the period of intermission, and it cannot be denied that this method is often followed by success. Still, where there can be certainty that the *simillimum* has been found, there can be no doubt the single dose is the best. Then, if this be followed by another paroxysm, if this be less severe than its predecessor, or is apparently modified curatively in any way, it will be perfectly safe to leave the dose given to finish the work it is so clear it has successfully begun.

Then as to form of the dose—shall it be given dry or in solution? After many years of careful observation of the action of medicines given in both forms, we have come to the conclusion which favors giving medicines in solution rather than in the dry form. Dissolved, they seem to act more promptly and pervadingly.

Quantity, as predicated of the dose of the properly selected curative, it may be remembered that error in this is much more likely to be found on the side of too much than too little, and, more than this, that the error of a wrong selection can never be rectified or compensated by any increase of dose of the wrongly selected drug. Hahnemann was wholly right when he said the dose of the rightly selected, *i. e.*, homœopathic remedy, was to be the smallest possible. If we were to suggest an improvement on this, it would be that all ideas of *large* and *small*, as to the dose, be dismissed from the thoughts of the prescriber, as these can be of no possible service to him or his patient. Large and small can hardly be predicated of that which is wholly dynamic. And then, further, that violence of symptoms call only for greater care in selecting the curative, and never for more of the drug in which this is found.

### PART II.—REPERTORY.

TRANSLATED FROM BŒNNINGHAUSEN.

### Repertory of the Combinations of the Elements of Paroxysms.

*Beginning with Chill.*

*Chill then heat.* Acon., Alum., Ant-tart., Arn., Bell., Carbo-v., Chin., Cina, Cycl., Dros., Graph., Hep., Hyos., Iod., Ign., Ipec., Lyc., Magn., Natr., Natr-mur., Nux-v., Petr., Phos., Puls., Rhus, Sabad., Secale, Spig., Spong., Stram., Sulph., Valer. or Ambr., Amm., Amm-mur., Ant-c., Apis, Ars., Asar., Bar., Bor., Bry., Calc., Canth., Carbo-an., Caust., Cham., Coff., Creos., Croc., Dig., Dulc., Guai., Hell., Kali, Lach., Laur., Mag-mur., Merc., Merc-corr., Nitr., Nit-ac., Nux-m., Op., Phos-ac., Scill., Sep., Staph., Thuja, Verat.

*Chill, then sensation of heat.* Merc-cyan., Sulph.

— — *heat in single parts.* Cycl.

— — *heat in face.* Ambr., Nux-v., Petr. or Acon., Chin., Creos., Cycl., Kali, Op., Stann.

— — *heat in the head.* Ipec.

— — *heat with thirst.* Acon., Bell., Hep., Puls., Rhus, or Bor., Chin., Dros., Merc., Secale, Spig., Sulph.

— — — — *without thirst.* Spig. or Amm-mur., Bor., Canth., Cina, Coff., Nux-m., Phos-ac., Rhus, Sulph.

— — — — *without sweat.* Sep.

— *with thirst, then heat.* Cina.

— — *then heat without thirst, then sweat.* Ign.

— — *then heat with thirst, then sweat.* Spong.

— *without thirst, then heat with thirst.* Petr.

— — *then heat with, then sweat without, then heat with thirst.* Ant-c.

— — *then heat without thirst.* Ars., Cycl., Dros., Hell., Phos-ac.

*Chill, then heat, and thirst with both.* Ant-c.
— *then heat, then chill with thirst.* Sulph.
— *then heat, then sweat.* Ars., Bry., Chin., Graph., Ign., Ipec., Lach., Natr-mur., Nux-v., Puls., Rhus, Sabad., Spong., Sulph., Verat. or Amm., Amm-mur., Apis, Bell., Bov., Caps., Carbo-an., Carbo-v., Caust., Cham., Cina, Cocc., Dig., Dros., Hep., Kali, Lyc., Mag-mur., Natr., Nitr-ac., Op., Plumb., Sabin., Samb., Sep., Staph., Thuja.
*Chill, then heat alternating with thirst, then sweat.* Sabad.
— *then heat, then sweat with thirst.* Rhus.
— — *then sweat without thirst.* Amm-mur., Nitr-ac.
— — *then sour sweat.* Lyc.
— *then heat with sweat.* Acon., Bell., Cham., Chin., Nux-v., Op., Puls., Rhus, Sabad. or Ant-tart., Bry., Caps., Carbo-v., Cina, Dig., Graph., Hell., Hep., Ign., Kali, Mezer., Nitr-ac., Phos., Spig., Sulph.
— — *without sweat.* Graph., Natr-mur.
— — *with sweat on the face.* Alum.
— — *with internal chill, then heat and sweat.* Phos.
— *with, then heat without thirst.* Hep. or Nitr.
— — — *then sweat.* Kali.
— *with heat.* Acon., Arn., Ars., Bell., Calc., Cham., Cocc., Coff., Dig., Hell., Ign., Merc., Mezer., Nitr-ac., Nux-v., Par., Petr., Plumb., Puls., Rhus, Scill., Sep., Spig., Sulph., Thuja, Verat, Zinc. or Alum., Amm., Anac., Ant-tart., Bar., Bov., Bry., Camph., Canth., Carbo-v., Chin., Cina, Colch., Coloc., Euphorb., Graph., Iod., Kali, Lach., Lyc., Mosch., Natr., Oleand., Phos., Phos-ac., Plat., Ran-bulb., Sabad., Samb., Selen., Sil., Spong., Vit.
— — *with external heat.* Anac., Arn., Ars., Bell., Calc., Cocc., Coff., Dig., Hell., Ign., Laur., Merc., Nux-v., Ran-bulb., Scill., Sep., Sil., Thuja or Acon., Bry., Chin., Colch., Lach., Lyc., Merc., Mezer., Nitr., Phos., Plumb., Rheum., Selen., Sulph., Vit.
— — *with flying heat.* Puls. or Ars., Merc., Plat.
— *with internal heat.* Acon., Ars., Bry., Cham., Euphorb., Ign., Kali, Mezer., Mosch., Puls., Rhus, Scill., Sulph.,

Verat., Zinc. or Arn., Bell., Calc., Chel., Chin., Hell., Iod., Merc., Nitr-ac., Nux-v., Oleand., Phos-ac., Sabad., Secale, Spong., Stann.

*Chill, with internal heat, with sensation of heat.* Oleand.

*Chill with internal heat and thirst.* Calc., Kali.

*Chill with heat, both internal.* Petr.

— *of single parts with heat of other parts.* Ign., Rhus or Chin., Nux-v., Sabad.

— — *with heat without sweat.* Sulph.

— — — *with sweat.* Verat. or Nux-v.

— — — *then sweat.* Graph.

— *then sweat, without previous heat.* Bry., Caps., Carbo-an., Caust., Clem., Dig., Lyc., Mezer., Nitr., Petr., Rhus, Thuja, Verat. or Amm-mur., Carbo-v., Cham., Chel., Hell., Hyos., Merc., Merc-corr., Nat-mur., Nux-v., Op., Phos., Phos-ac., Sabad., Sep., Spig.

— *then cold sweat.* Verat. or Ars.

— *then sweat without heat and thirst.* Amm-mur., Bry., Caust.

— *then sweat, then heat.* Bell.

— — *then thirst.* Lyc.

— *then thirst, then sweat.* Thuja.

— *alternating with heat.* Ambr., Amm-mur., Ant-tart., Ars., Asar., Bar., Bell., Bry., Calc., Cham., Chin., Cocc., Coloc., Creos., Dig., Hep., Hyos., Lach., Lyc., Merc., Nux-v., Phos., Phos-ac., Rhus, Selen., Sep., Spig., Verat., Vit., Zinc. or Amm., Arn., Aur., Bor., Canth., Caust., Chel., Coff., Dros., Graph., Iod., Kali, Laur., Led., Mosch., Nat-mur., Nitr., Nit-ac., Rheum., Rhod., Sabad., Samb., Sil., Stram., Sulph., Valer.

— — *then heat.* Verat.

— — *then sweat.* Kali or Bry., Spig.

— *alternating with sweat.* Euphorb., Verat. or Ars., Calc., Led., Lyc., Nux-v., Puls., Sabad., Sulph., Thuja.

BEGINNING WITH HEAT.

*Heat, then chill.* Bell., Bry., Calc., Caust., Hell, Nit-ac., Nux-v., Sep., Stann., Staph., Sulph. or Apis., Ang., Calad.,

Caps., Chin., Dulc., Ign., Lyc., Men., Merc., Petr., Phos., Puls., Thuja.

*Heat, then chill, then heat, then sweat.* Rhus.

— *then coldness.* Nux-v. or Calc., Caust., Sep., Sulph.

*Heat, then shuddering.* Ang., Sulph. or Caps., Cocc., Hell., Natr-mur., Puls., Rhus.

— *of the face, then shuddering.* Sulph.

— *of the head, then coldness, then heat.* Stann.

— *then sweat.* Amm-mur., Ars., Carbo-v., Cham., Chin., Coff., Ign., Ipec., Nux-v., Ran-scel., Rhus, Sil., Verat. or Ant-c., Ant-tart., Bell., Bor., Bry., Calc., Carbo-an., Cina, Creos., Graph., Hell., Hep., Lach., Lyc., Nit-ac., Op., Pet., Puls., Rhod., Spong., Staph., Stront., Sulph.

— *then cold sweat.* Verat or Caps.

— *then sweat, then thirst.* Calad.

— — *then heat.* Ant-c.

— *with shuddering chill.* Bell., Cham., Hell., Nux-v., Rhus, Zinc. or Acon., Arn., Asar., Bry., Coff., Ign., Merc., Mosch., Puls., Sep., Spig., Sulph.

— *with shuddering chill and thirst.* Caps.

— — *without thirst.* Hell. or Spig.

— — *then sweat.* Caps.

— *with internal chill.* Anac., Bell., Ign., Lach., Nux-v. or Acon., Ars., Bry., Calc., Coff., Laur., Lyc., Men., Merc., Nitr., Phos., Sep., Sil., Thuja.

— *with external coldness.* Ars., Bell., Calc., Mezer., Sabad., Verat. or Arn., Bry., Chin., Euphorb., Hell., Iod., Merc., Mosch., Phos., Phos-ac., Puls., Rhus, Spong., Stann.

— *with coldness of single parts.* Nux-v. or Chin., Ign., Rhus.

— *with sweat.* Bell., Calc., Caps., Cham., Con., Hell., Hep., Ipec., Natr., Nux-v., Op., Rhus, Sabad., Sarsap., Stann., Staph., Stram., Sulph-ac., Valer., Verat. or Acon., Alum., Amm-mur., Bry., Chin., Cina, Euphorb., Ign., Laur., Merc., Nitr-ac., Par., Phos., Plumb., Puls., Sep., Spig., Spong., Sulph., Tar., Thuja, Viol-tr.

— — *and thirst.* Con., Hep., Merc., Rhus.

*Heat, with sweat, without thirst.* Amm-mur., Bell., Bry., Caps., Hep., Nux-v., Phos., Spig., Stram., Verat.

*Heat with sweat and thirst, then coldness.* Stann.

— *and thirst alternating with chill.* Calc.

— *with thirst then sweat.* Coff.

— *in the head alternating with coldness of the legs.* Sep., Stann.

— *alternating with shuddering.* Bry., Cocc., Ipec., Plat. or Acon., Lach., Mosch., Phos-ac. (See shuddering alternating with heat.)

— *with sweat, then chill.* Stann.

— — *external coldness, then chill, then heat with external coldness.* Phos.

— *alternating with sweat.* Amm-mur., Chin., Led., Samb. or Bell., Colch., Natr., Nux-v., Sep., Sulph.

BEGINNING WITH SHUDDERING.

*Shuddering then chill.* Bry. or Ars., Ipec., Lach.

— — *without thirst.* Ipec.

— — *then heat without sweat.* Ars.

— *then heat.* Bell., Ign., Sep., Sulph. or Ang., Apis, Asar., Bry., Canth., Carbo-v., Cocc., Con., Gels., Graph., Lach., Laur., Led., Mosch., Nux-v., Sil., Staph.

— — *with chill.* Bry., Lach.

— — *with thirst.* Con., Sulph.

— *then sweat.* Clem., Natr-mur., Rhus or Bry., Caps., Caust., Dig., Graph.

— *with heat.* Bell., Cham., Hell., Nux-v., Rhus, Zinc. or Acon., Anac., Arn., Ars., Asar., Bry., Calc., Caps., Coff., Dros., Ign., Kali, Merc., Mosch., Puls., Rheum., Sep., Spig., Sulph.

— *with heat of face, without thirst.* Anac., Ars., Calc., Dros., Kali.

— *with sweat.* Rhus. or Acon.

— *alternating with heat.* Ars., Bell., Bry., Cham., Cocc., Merc., Nux-v., Plat., Sep. or Acon., Amm., Asar., Bor., Calc., Caust., Chel., Chin., Coff., Coloc., Graph., Hep., Kali,

